# Intelligent Force-Measurement System Use in Shock Tunnel

**DOI:** 10.3390/s20216179

**Published:** 2020-10-30

**Authors:** Yunpeng Wang, Zonglin Jiang

**Affiliations:** 1 State Key Laboratory of High Temperature Gas Dynamics, Institute of Mechanics, Chinese Academy of Sciences, Beijing 100190, China; zljiang@imech.ac.cn; 2 School of Engineering Science, University of Chinese Academy of Science, Beijing 100049, China

**Keywords:** artificial intelligence, deep learning, dynamic calibration, force-measurement system, strain-gauge balance

## Abstract

The inertial vibration of the force measurement system (FMS) has a large influence on the force measuring result of aircraft, especially on some tests carried out in high-enthalpy impulse facilities, such as in a shock tunnel. When force tests are conducted in a shock tunnel, the low-frequency vibrations of the FMS and its motion cannot be addressed through digital filtering because of the inertial forces, which are caused by the impact flow during the starting process of the shock tunnel. Therefore, this paper focuses on the dynamic characteristics of the performance of the FMS. A new method—i.e., deep-learning-based single-vector dynamic self-calibration (DL-based SV-DSC) of an impulse FMS, is proposed to increase the accuracy of aerodynamic force measurements in a shock tunnel. A deep-learning technique is used to train the dynamic model of the FMS in this study. Convolutional neural networks with a simple structure are applied to describe the dynamic modeling so that the low-frequency vibration signals are eliminated from the test results of the shock tunnel. By validation of the force test results measured in a shock tunnel, the current trained model can realize intelligent processing of the balance signals of the FMS. Based on this new method of dynamic calibration, the reliability and accuracy of force data processing are well verified.

## 1. Introduction

**Figure 1:**
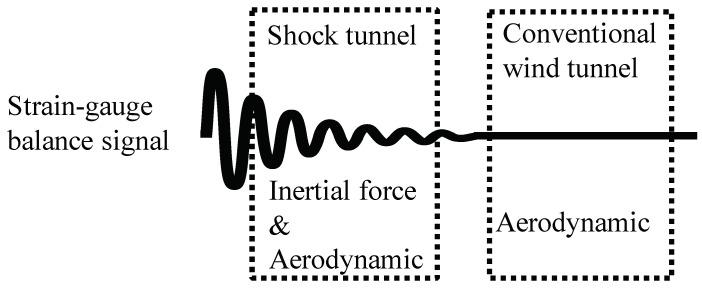
Balance signal processing in a shock tunnel and the conventional wind tunnel.

Aerodynamic force measurement in a high-enthalpy flow is very important for the design and optimization of hypersonic vehicles. Currently, impulse facilities are used to generate the high-temperature and high-pressure driving gas needed to simulate high-enthalpy flow with hypersonic flight conditions, such as in a shock tunnel. However, when the force tests are conducted in an impulse facility, the inertia has a large influence on the measured results (see Figure 1). For a conventional hypersonic shock tunnel, owing to the instantaneous flow and short test time (generally 500 μs–20 ms) [1,2,3,4], mechanical vibration of the model-balance-support (MBS) system occurs, which leads to low-frequency vibrations of the test model and its motion that cannot be addressed through digital filtering. For the MBS system, the lowest natural frequency of 1 kHz is sometimes required for a test time of typically 5 ms to obtain improved measurement results [2]. The higher the natural frequencies are, the better the justification for the neglected acceleration compensation. For these test conditions, many researchers have proposed several special balances to measure the aerodynamic forces in impulse facilities—i.e., the accelerometer balance [5,6,7], the stress-wave force balance [8,9,10], the free-flight measurement technique [11,12,13,14,15], the compensated balance [16], and the impulse-type strain gauge balance (SGB) [17,18]. Owing to the very short test time, however, mature technology has not been developed for the force measurement in a shock tunnel.

Although research on the static characteristics of strain-gauge balance is very mature, there are very few studies on its dynamic characteristic analysis and dynamic correction because of the special application and few dynamic testings in traditional wind tunnels. The traditional research methods for the dynamic characteristics include dynamic compensation, dynamic decoupling, and frequency dynamic correction to improve the dynamic characteristics of sensors. At present, the common dynamic force tests in a wind tunnel mainly include the rolling missile test [19], model dynamic stability derivative tests [20,21,22,23,24], and shock tunnel test [8]. The current work focuses on the balance design and the high-precision force tests in an impulse facility, such as a shock tunnel. Actually, the limitation of the traditional strain measurement technology is very difficult to overcome in a short-duration force test because of the influence of inertial vibration. Therefore, the dynamic calibration becomes very important for improving the precision and accuracy of the force measurement during the short test time. In the conventional dynamic calibration for a force measurement system or balance system, an approximate step change in different loads on the model can be applied by attaching a fine wire to the tip of the model, applying a load to it, and then cutting the wire [10]. The value of the step load is changed by choosing different weights. Then, a dynamic model can be obtained by analyzing the impulse responses of the balance system. Although this kind of dynamic calibration method is widely used, it has many problems that effect its measurement accuracy. First, the direction of the step load is very difficult to accurately control and measure, which will cause some calibration errors. Second, changing the weights becomes tedious and time consuming, especially for six-component loading. Third, the traditional technique makes it easy to apply a step load to a single component, but it is very difficult to load multiple components simultaneously.

A new method—i.e., the single-vector dynamic self-calibration (SV-DSC) of the impulse force measurement system (FMS)—is proposed in this paper for obtaining the accurate aerodynamic force in a shock tunnel with short duration. In this method, a deep-learning (DL) technique—i.e., the convolutional neural network (CNN), is used in the dynamic calibration, where a learning algorithm based on several layers of neural networks is applied to describe and build the dynamic model of the FMS.

## 2. Concept of Intelligent Force-Measurement System

**Figure 2:**
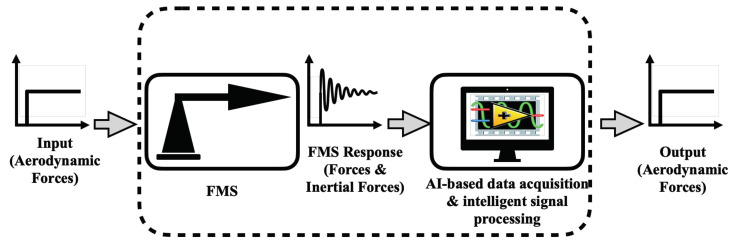
Signal processing of intelligent force-measurement system.

Based on artificial intelligence (AI) technique, an intelligent force-measurement system (iFMS) can be constructed and applied to high-accuracy dynamic force tests in the shock tunnel with very short-duration. Figure 2 shows the structure of iFMS.

The iFMS includes a test model, an impulse strain-gauge balance, and a sting support system. Among them, the impulse force balance is iFMS’ core measuring sensor. The SGB has to be designed for the complex structures based on a number of influencing factors, such as the starting process of impulse facility, mass and aerodynamic characteristics of the test model, structural characteristics of the sting support, etc. Therefore, the concept of iFMS includes the designs of the test model, impulse balance, and sting structure, but also contains two key techniques, namely the static- and dynamic-calibration processes. During the calibration process, it is necessary to perform the accurate static-calibration for the FMS’ sensor (a SGB). After the static-calibration of SGB, the high-precision dynamic calibration is performed for impulse iFMS used in the shock tunnel. A “single-vector loading” technique is proposed and employed in the present dynamic-calibration of the iFMS. The calibration method is the DL-based SV-DSC technique. The object of dynamic-calibration is for the overall system of FMS, not just for the SGB. The calibrated FMS (iFMS) will be used as it is in the force tests. Using deep-learning technique, the data obtained by SV-DSC are pre-processed and trained by a sample CNN, where a learning algorithm based on several layers of neural networks is applied to describe and build the dynamic model of iFMS. Therefore, the iFMS can be used for intelligent processing of force measuring signals with inertial vibration.

## 3. Principle of Single-Vector Dynamic Self-Calibration

In this study, a high-precision dynamic calibration is performed for an impulse FMS used in a shock tunnel. The object of dynamic calibration is the overall FMS, not just the strain-gauge balance. The calibrated FMS is used as it is in the force tests. The calibration method is the DL-based SV-DSC technology proposed in this study. Before the dynamic calibration, it is necessary to perform accurate static calibration for the FMS sensor (a strain-gauge balance). A balance calibration facility should be used in this process, which is a static calibration study; no further tautology will occur in this paper.

### 3.1. What Is SV-DSC?

**Figure 3:**
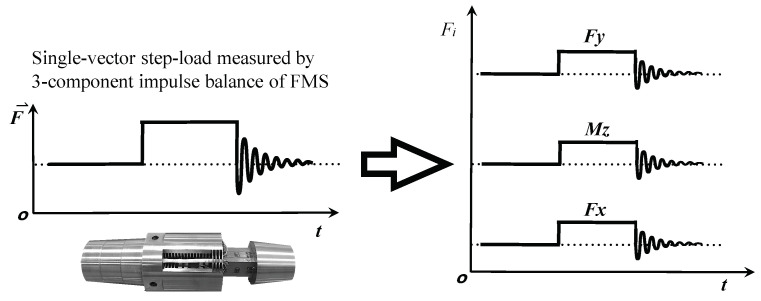
A single-vector load generates three-component step loads measured by the balance of the FMS.

**Figure 4:**
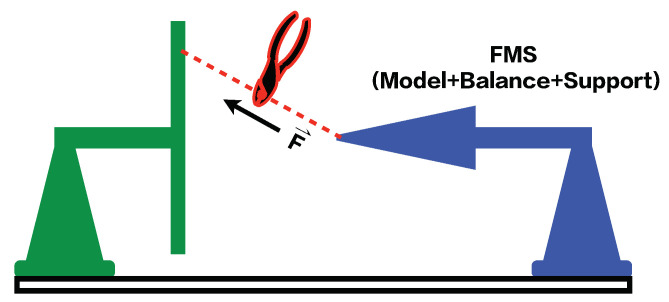
Schematic diagram of the step loading of SV-DSC method.

The dynamic calibration system consists of the following parts: a steel wire and its suspension structure, the FMS, and a data acquisition system (DAS). Among them, the FMS includes a test model, an impulse SGB, and a sting support system. A “single-vector loading” technique is proposed and employed in the present dynamic calibration. For the FMS actually used in the force test, a vector load is applied to the test model through a wire along any direction and then cut to achieve a step load. If the SGB is multi-component (three-component or six-component), multi-component loading and unloading can be realized, and the load value is output by the high-accuracy balance of the FMS in real time. The vector load is decomposed into multi-component loads in accordance with the coordinate system of the SGB during the static calibration process. Figure 3 shows the three-component step-loads that can be generated by a single-vector load $\vec{F}$ and the values of the step loads, which can be measured in real time by the impulse SGB. The above operation is called “self-calibration” in this dynamic calibration method. Figure 4 shows a schematic diagram of the single-vector step-loading procedure.

The SV-DSC method does not use weights with fixed direction for loading but still uses the traditional method of instantaneous unloading to apply a step load. The wire (generally a thin steel wire with a diameter of less than 0.5 mm) is used to apply a single-vector load $\vec{F}$ and yields a step load when it is cut. Therefore, with the SV-DSC method, the multi-component step loads can act on the test model at the same time and be measured more accurately by the impulse balance of the iFMS. This load mode is closer to the actual state of the impulse flow through the test model during a shock tunnel run. The main performance of SV-DSC depends on the accuracy and precision of the SGB by the static calibration, while the traditional weight-loading method is greatly affected by the wire direction and the accuracy of the weight. The wire direction with a load is generally difficult to accurately match to the selected coordinate system.

In addition, SV-DSC is convenient, and the device is simple and easy to use. When performing dynamic calibration, the iFMS can be located inside or outside the shock tunnel, which ensures that the iFMS can be directly used for the force test in the shock tunnel. In the traditional dynamic calibration, the dynamic calibration is mainly focused on the wind tunnel balance. Therefore, the objects in the traditional dynamic calibration and in the force test are very different in terms of the FMS structure or are even completely different. As is known, the vibration characteristics (modal frequencies) of a solid body are directly related to its mass, structure, and material properties. Thus, a dynamic calibrated balance (or similar structure of the iFMS) with high precision may still produce a large measurement error in the force test because of the different structure of the iFMS.

### 3.2. Deep-Learning-Based Dynamic Modeling for Vibration Feature

Actually, in many fields, the deep-learning technique has been applied and has solved many key technical problems. For example, Antczak [25] presents a novel approach to denoise electrocardiographic signals with deep recurrent denoising neural networks. Chiang et al. [26] also proposed a denoising autoencoder using the fully convolutional network for electrocardiogram signal denoising. In this study, the postprocessing of the collected feature signals does not involve the method of system identification but introduces a deep-learning technique—i.e., it uses the convolutional neural networks to model the dynamic feature of the measured signal. Convolutional networks have played an important role in the history of deep learning. Convolutional networks [27], also known as convolutional neural networks, are a specialized kind of neural network for processing data that have a known, grid-like topology [28]. Examples include time-series data [29,30], which can be thought of as a 1D grid taking samples at regular time intervals, and image data, which can be thought of as a 2D grid of pixels. Convolutional networks have been tremendously successful in practical applications [31,32,33]. The name “convolutional neural network” indicates that the network employs a mathematical operation called convolution. Convolution is a specialized kind of linear operation.

In this dynamic calibration study, the basic idea is to use the ideal step load as an output training sample and a dynamic signal with inertial vibration interference as an input training-sample to train the AI model, which can describe the FMS vibration features. It is also applied to the test data processing of the same FMS, which is also an inertial compensation of the balance signal based on the CNN trained model.

#### 3.2.1. Modeling Training Process

The process of dynamic modeling by the SV-DSC method is as follows.

**Step** **1** The iFMS is built to assemble the SGB, test model and sting support. Before the dynamic calibration of the iFMS, a high-precision static calibration of the SGB is required. The SGB is a single-component, three-component, or six-component SGB, which is determined according to the test requirements.
**Step** **2** At the front of the test model, a loading/unloading mechanical device is placed, which is designed as a “ten” fixed support, and a wire fixing point is set in a small slot. Thus, a step load can be applied to the model through this device and the wire.
**Step** **3** On the windward surface of the model, according to the calibrated load requirement, the corresponding suspending points of the wire are set.
**Step** **4** The balance output of the iFMS is recorded by the DAS before suspending the wire, and the data are used as the zero-load sample.
**Step** **5** One end of the wire is connected to the selected suspending point on the test model, and the other end is fixed to a point on the wire support on the step loading device. At this time, a single-vector force $\vec{F}$ acts on the test model. Its direction is along the pull wire, and the value can be measured and output by the balance system.
**Step** **6** By cutting the pull wire at one end of the step-loading device, the unloading process of the single-vector $\vec{F}$ is completed. At this time, the signals output by the iFMS are the multi-component step loads, which are recorded by the DAS in real time, and the extraction process of one sample data point for SV-DSC is completed.
**Step** **7** After obtaining the test sample of the step load, the data should be preprocessed. Because of the difference in data dimension and value range, the sample data point is normalized to change its value between −1 and 1. During testing and verification, it can be denormalized again.
**Step** **8** CNNs are used to perform deep-learning training on the processed input and output samples, and the output sample (ideal step load) is used as the train target during the training.

After the above model, the training process is completed; a CNN model can be obtained and used to eliminate interference signals caused by inertial vibration.

#### 3.2.2. CNN Learning Algorithm

Typically, the most expensive part of convolutional network training is learning the features. The output layer is usually relatively inexpensive due to the small number of features provided as an input to this layer after passing through several layers of pooling [28]. In the training of dynamic calibration data samples, CNNs adopt a block combination (this study initially uses only one block to achieve better results). Each block contains multiple convolutional layers, the size of which continuously increases (that is, the number of channels increases), and is used to extract vibration features from a subtle part to a wider part. Finally, the channel number of the original input data is restored to complete the fusion of information between multiple dimensions of the balance data.

**Figure 5:**
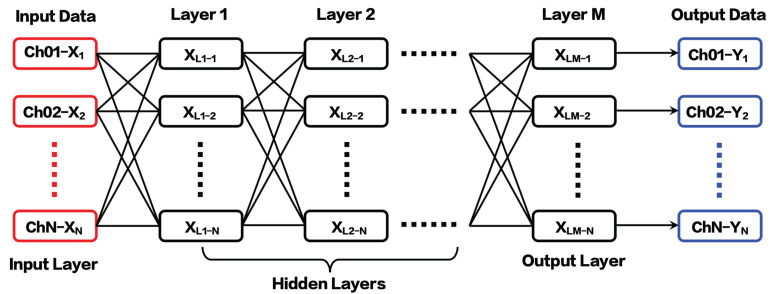
CNN learning algorithm for deep learning.

In the present study, the one-dimensional CNN is used in the training. The CNN structure used in the deep-learning training is shown in Figure 5. The input data on the left are the step-load signal with inertial vibration interference. Here, *N* is the number of components of the balance; in this study, a three-component SGB is used, that is, *N* = 3. The output data on the right are the ideal step-load signal, and the number of channels must be consistent with the input data. The middle is the hidden layer; hence, three layers are used in the present training process. In the first layer of the current CNN model, the number of channels in the input signal is 3 and the number of channels produced by the convolution is 64, while the size of the convolving kernel is 50. In the second layer, the input and output channels are 64 and 64, respectively, while the corresponding kernel size is set to 30. As for the output layer, the input and output channels become 64 and 3, respectively, where a deconvolutional layer with a stride of 1 produces the three output signals. Therefore, the parameters L1−N and L2−N both take 64—that is, they expand the original three-channel data signal to a 64-channel signal. L3−N takes 3—that is, the 64-channel signal is eventually converted into a 3three-channel signal and output. Additionally, in the present training, only a simple one-dimensional CNN is used for modeling.

To compare with different networks with different number of layers (such as four, five, and six layers), the three-layer model can effectively capture the vibration characteristics of the balance signal in the force measurement system. In this method, one of the basic ideas is that the CNN model must be simple enough so that it can be easily applied to engineering.

### 3.3. Calibration Device and Data Acquisition

**Figure 6:**
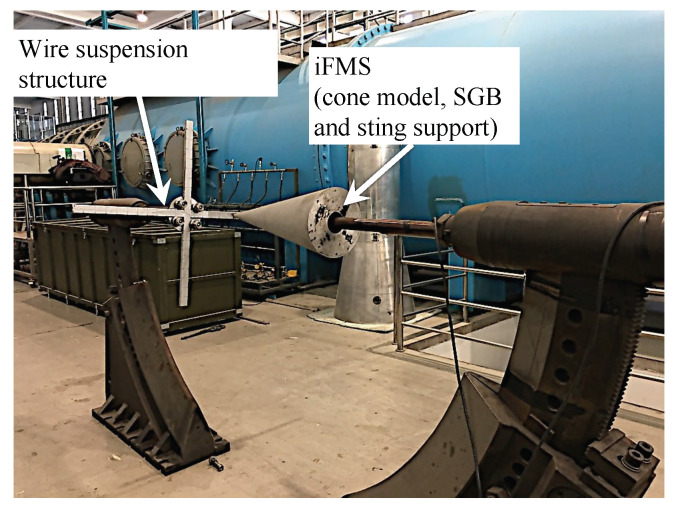
Photo of calibration device for SV-DSC.

Figure 6 shows the calibration device system for the SV-DSC in the present study. A cone made of aluminum alloy with a length of 0.75 m and a $10^{\circ }$ semivertex angle was employed as a test model in the calibration test. The cone is a standard model and has a considerable amount of test data. The impulse balance, which uses a strain-gauge sensor, has three components—i.e., the normal force $F_{y}$, pitching moment $M_{z}$, and axial force $F_{x}$. The NI (National Instruments) high-performance DAS, with a PXIe-8880 controller and a PXIe-4331 eight-channel acquisition module, was used; its sampling rate is 102.4 kS/s, with a resolution of 24 bits. In the present dynamic calibration, a fine steel wire with a diameter of 0.5 mm is used due to its good stiffness and high strength, as these wire characteristics are very important for obtaining an approximately ideal step load.

During the dynamic self-calibration, the steel wire can be pulled and suspended at any position of the cross structure. With the current loading method, the efficiency of applying the step load is relatively high, and a set of calibration data can be acquired within one minute. During the sample collection processing, 7500 points (150 milliseconds) are recorded as a sample data, which includes the effective run time of 100 milliseconds in the long-duration shock tunnel (an impulse wind tunnel at Institute of Mechanics). In addition, the value of each single-vector load should be within the range of aerodynamic force, so that the training samples are closer to the real situation. This setting can improve the performance of the training model.

In the preliminary test, 120 sets of dynamic calibration data were obtained and used to train the dynamic model of the iFMS using the CNN. In practice, the amount of data is limited. However, based on the current training results, the amount of data seems to be acceptable, and satisfactory results are obtained. In the next study, the amount of data will be increased, and the data processing ability of the trained model will be evaluated in detail. The current preliminary work published in this paper is a feasibility study.

## 4. Test Signal Processing and Analysis

In the dynamic modeling, 120 sets of dynamic load signal samples are used for deep-learning training based on the time-domain features, which are divided into two parts: 100 sets are used to train the model, while the others are used to perform the verification test.

### 4.1. Test Verification

**Figure 7:**
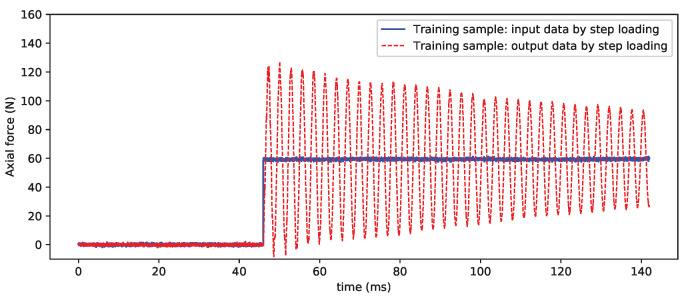
Ideal step loading (output data) and unloading (input data).

**Figure 8:**
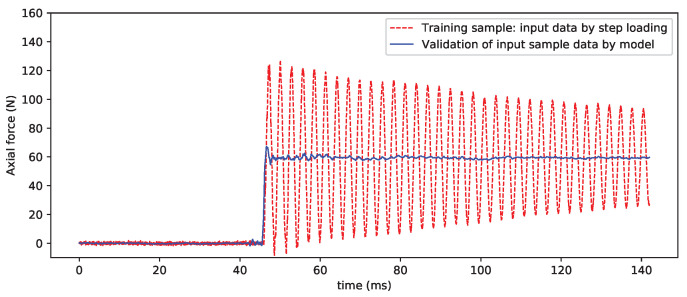
Input sample data and validation by CNN model (axial force).

Figure 7 compares the input and output signals used for CNN model training. The purpose of CNN model training is to eliminate the interference of the inertial vibration for the balance signals. Figure 8 shows the processing results of the sample vibration signal. It can be seen that the effect is very good and that the inertial vibration in the sample data curve is basically filtered out, which restores the “real” step load to some extent.

**Figure 9:**
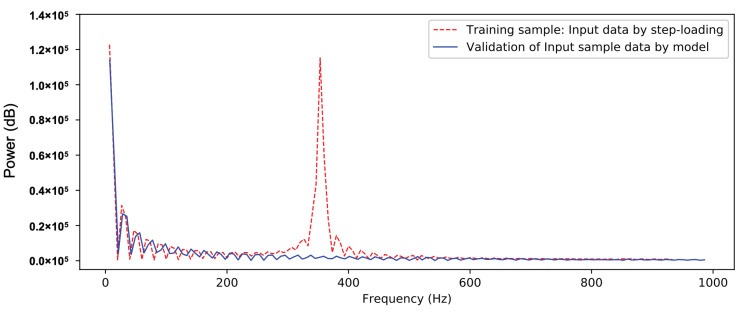
Data processing and comparison by FFT (axial force).

From a certain perspective, the data processing of the SV-DSC method can be regarded as a process of “intelligent filtering” for the inertial vibration signal. This process can be more clearly understood from the following spectrum comparison. In Figure 9, the fast Fourier transform (FFT) is performed on the vibration sample signal and the signal is processed by the CNN model. It can be clearly seen that the main frequencies in the unloaded oscillation signal are completely filtered out.

**Figure 10:**
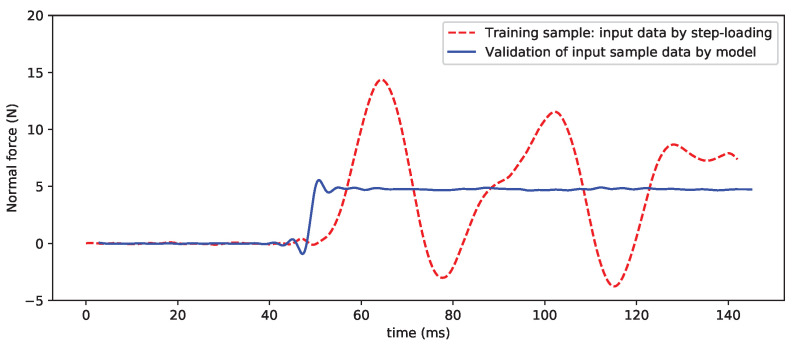
Input sample data and validation by CNN model (normal force).

**Figure 11:**
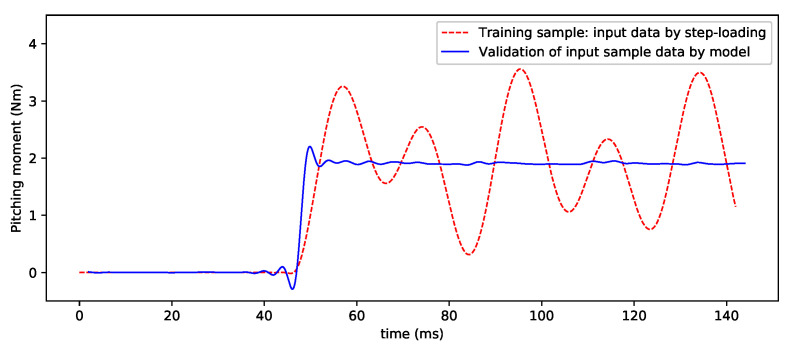
Input sample data and validation by CNN model (pitching moment).

For the verification of the other component data, Figure 10 and Figure 11 show the processed results of the normal force and pitching moment, and the preliminary results are good. However, the current methods in the deep-learning training and sample acquisition still require further improvement, as, for example, the noise interference in the small output of some components is relatively large. As the DL-based SV-DSC technique is further researched and improved, these problems will be solved very well.

### 4.2. Error Analysis

The central challenge in machine learning is that we must perform well on new, previously unseen inputs—not just those on which our model was trained. The ability to perform well on previously unobserved inputs is called generalization [28]. Typically, when training a deep-learning model, some error measured on the training set, which is called the training error, can be computed, and this training error can be reduced. To quantify the performance of the trained model on the training set and test set, the training error and test error are used, respectively; the latter is also often referred to as the generalization error. Therefore, a good model should have minimum training error and minimum generalization error at the same time; an algorithm with a good generalization ability can meet these requirements. In the study, the mean squared error (MSE) is used to evaluate the training error and test error—that is, to evaluate the performance of the neural network. The MSE reflects the error between the output and the expected output by the trained model, which is the stability of the model. The mean squared error is the average value of the square of the point-by-point error, given the following definition:

(1) $$MSE=\frac{1}{n}\sum_{i=1}^{n}{\left(R_{s}-R_{a}\right)}^{2}$$

where $R_{s}$ is the evaluation value, $R_{a}$ is the test value, and *n* is the number of data points. During the training, the difference between the obtained curve and the target curve is computed as an error.

**Figure 12:**
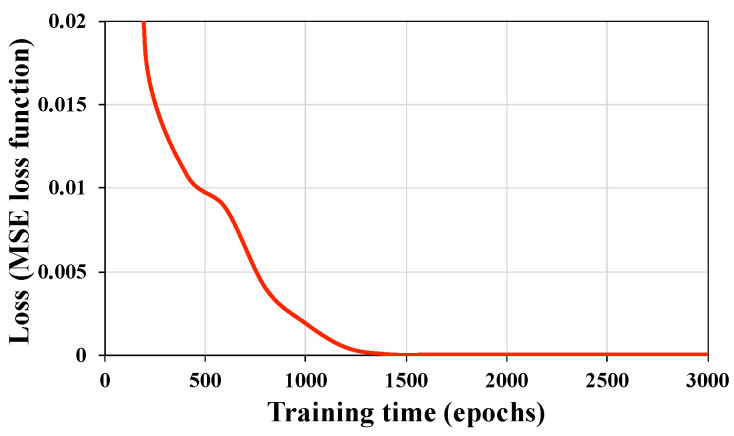
Training loss with time (epochs).

In Figure 12, a learning curve shows how the loss changes over time (indicated as the number of training iterations over the dataset, or epochs). After 1000 iterations (epochs), the MSE is 1.88 × 10${}^{-3}$. At this time, the CNN convolutional layer has begun to identify the core dynamic features, but the detailed ones that control the higher-order errors have not yet been fully obtained. After 2000 epochs, the MSE drops below 2 × 10${}^{-6}$. It can be considered that the CNN convolutional layer at this time has recognized the main part of the error in the dynamic calibration and that the structural vibration characteristics of the iFMS have been more accurately identified. Thus, an effective dynamic calibration process is completed by the DL-based SV-DSC device.

The verification sample $R_{vs}$ is processed by the model to obtain the evaluation data $R_{va}$, and then the intercepted data (5000 points per channel, approximately 80 ms) is averaged and $\bar{R_{vs}}$ and $\bar{R_{va}}$ are obtained. The relative error Err can be directly calculated by the following formula:

(2) $$E_{rr}=\frac{\bar{R_{vs}}-\bar{R_{va}}}{\bar{R_{va}}}\times 100\%$$

It can be seen from the data in Table 1 that the model established by deep learning is very good for the intelligent processing of signals with vibration interference. For the data of the normal force with a small output, the relative error can reach approximately 5‰, and the axial force can reach 1‰. This result is consistent with the balance performance achieved by the static calibration—that is, the accuracy of the axial force is also higher than in the cases of the normal force and the pitching moment. Therefore, the accuracy of SV-DSC depends on the accuracy achieved by the static calibration.

In this study, by dynamic modeling of the three-component iFMS, the deep-learning model can be used to process the test results measured by the iFMS to further verify the feasibility and effectiveness of SV-DSC. The purpose of this study is to build an intelligent measuring system to realize the intelligibility of test signal processing.

## 5. Application of SV-DSC in Shock Tunnel Tests

**Figure 13:**
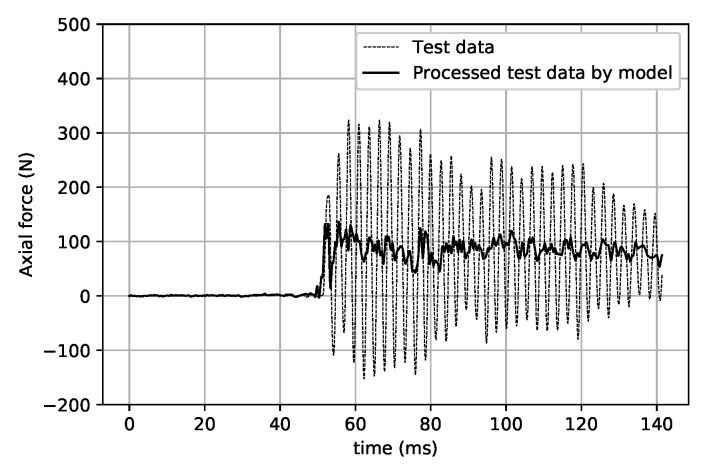
Test data and their processing by the model (axial force).

**Figure 14:**
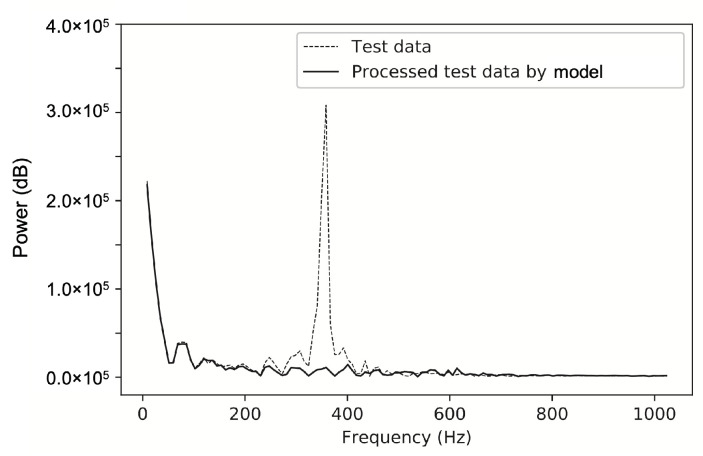
Test data processing and comparison by FFT (axial force).

In the force test validation, the balance signals in the force test of a 750 mm cone in the JF-12 shock tunnel were reprocessed by the same CNN model. From the axial force results in Figure 13, it can be seen that the CNN model ideally “filters out” the large-scale inertial vibration effects and gives the results of the axial load with a step-load-like feature. The results of the two signals obtained by FFT processing (see Figure 14) clearly show that the signal with a vibration frequency of approximately 380 Hz is basically filtered out.

**Figure 15:**
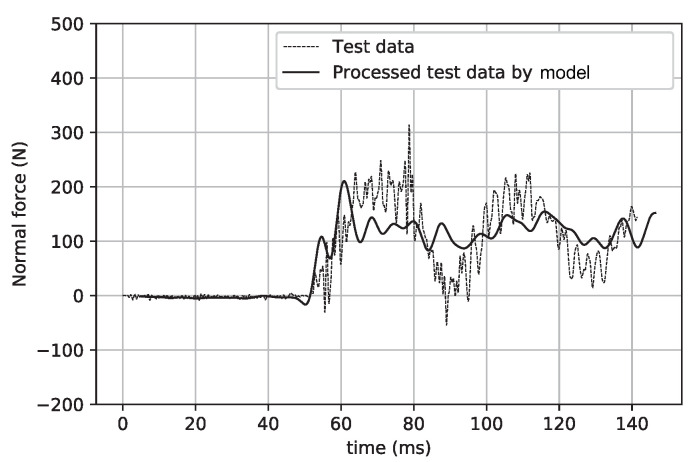
Test data and their processing by the model (normal force).

**Figure 16:**
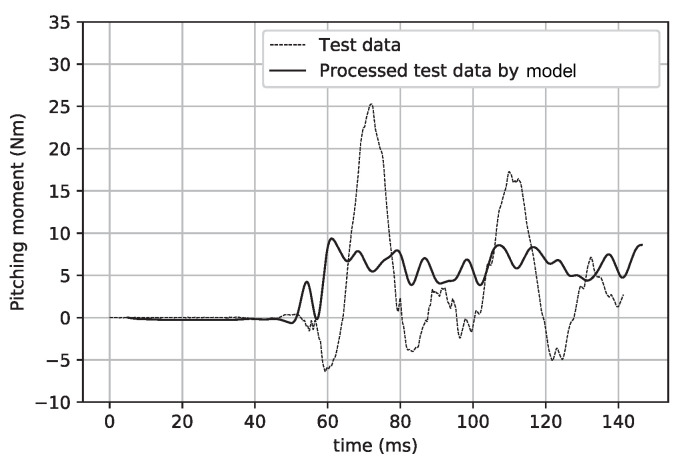
Test data and their processing by the model (pitching moment).

Figure 15 and Figure 16 show a comparison of the signals before and after the intelligent processing by the CNN model in the cases of the normal force and pitching moment. Obviously, the large-scale inertial vibration interference is eliminated by the CNN model processing. Table 2 shows the data deviation achieved by the direct average method and CNN model. In these results, the test data were intercepted within the time coordinate range of 70–80 ms for average processing. In addition, the test data that underwent direct average processing were analyzed for errors in the previous work, and the precision of these data was higher than 2% [17,18].

From the results in Table 2, it can be seen that there is a deviation of approximately 1 to 2% between the two data processing methods. One of the main reasons for this deviation is due to the inertial interference caused by the large-scale vibration of the force measurement system. On the basis of the comparison and analysis, the data processed by the current deep-learning models have higher reliability.

In addition, the data processed by the deep-learning model clearly show the unfiltered vibration signals, which are obviously not current noise. The main influencing factors include the flow noise, the nonuniformity of flow in the wind tunnel, and the very small balance signal (the load is relatively small). These factors may cause large errors during the processing of data, especially during the direct average processing.

## 6. Summary

To achieve a high-performance force measurement with short duration in an impulse facility, based on the fundamental influencing factors of the test performance, dynamic calibration highlights its importance and necessity, in addition to high-precision static calibration. In this paper, an intelligent force measurement system is presented. The intelligence of iFMS is mainly for the intelligent processing of the measuring signals. This function is realized by dynamic calibration of the iFMS based on AI technology. The single-vector dynamic self-calibration of a force measurement system using a deep-learning technique, is presented and used in the tests. By CNN modeling of the three-component FMS, the deep-learning AI model can be used to process the test results measured by the FMS to further verify the feasibility and effectiveness of SV-DSC. The purpose of this study is to build an intelligent force measurement system to realize intelligent processing of the test data measured in the shock tunnel. The DL-based SV-DSC method has the following characteristics.

1. The current method does not use the traditional weight but directly reads the value of the load along the wire by the strain-gauge balance (at the same time, it is directly decomposed into multi-component loads by this balance) and unloads by cutting the wire.
2. A single-vector load in any direction can realize a multi-component step load at the same time and accurate quantitative loading, which is closer to the state of aerodynamic loading in the actual force test.
3. The present method is convenient, and the device is simple and easy to use. The FMS can be placed outside the wind tunnel for the dynamic calibration and can be calibrated by SV-DSC directly in the test section of the wind tunnel. This ensures that the calibrated system is the FMS adopted for the force test.
4. The traditional dynamic calibration method mainly performs calibration on the force balance. However, in addition to the balance, the actual FMS also includes the test model and the support system. Therefore, the object of the traditional dynamic calibration is different from the test object in the structure, even completely different.

By validation of the test results, the current trained model can realize intelligent processing of the balance signals of the iFMS. For the data of the normal force with a small output, the relative error can reach approximately 5‰, and the axial force can reach 1‰. Comparison of test results in wind tunnel showed a deviation of approximately 1 to 2% between the averaged data and original data processed by simple CNN model. Based on deep learning-based dynamic calibration, the reliability and accuracy of force data processing are well verified.

In addition, the SV-DSC technique makes it easy to apply step loads with multi-component loads. An SGB with conventional structures can also be used for force measurement in an impulse facility with short duration, as the inertial force interference is reduced or eliminated by the SV-DSC technique. We believe that the future technique development of wind tunnel balance is the intelligibility of signal processing of the iFMS.

## Figures and Tables

**Table 1:** Validation error of three-component (channel) data.

Component	$\bar{R_{\mathrm{vs}}}$	$\bar{R_{\mathrm{va}}}$	Err
Normal force	0.00256	0.0025	0.56%
Pitching moment	0.1509	0.1501	0.53%
Axial force	0.02297	0.0229	0.10%

**Table 2:** Comparison of the test data processing carried out by the direct average method and the CNN model.

Aerodynamic Coefficient	Averaged Data	Data Processing by CNN Model	Deviation
Normal force	0.1580	0.1552	−2.21%
Pitching moment	0.1079	0.1067	−1.95%
Axial force	0.1026	0.1075	1.48%
